# The Potential Innovative Use of Bacteriophages Within the DAC^®^ Hydrogel to Treat Patients With Knee Megaprosthesis Infection Requiring “Debridement Antibiotics and Implant Retention” and Soft Tissue Coverage as Salvage Therapy

**DOI:** 10.3389/fmed.2020.00342

**Published:** 2020-07-31

**Authors:** Tristan Ferry, Cécile Batailler, Charlotte Petitjean, Joseph Chateau, Cindy Fevre, Emmanuel Forestier, Sophie Brosset, Gilles Leboucher, Camille Kolenda, Frédéric Laurent, Sébastien Lustig

**Affiliations:** ^1^Service des Maladies Infectieuses et Tropicales, Hôpital de la Croix-Rousse, Hospices Civils de Lyon, Lyon, France; ^2^Université Claude Bernard Lyon 1, Lyon, France; ^3^Centre Interrégional de Référence pour la Prise en Charge des Infections Ostéo-Articulaires Complexes (CRIOAc Lyon), Hospices Civils de Lyon, Lyon, France; ^4^CIRI—Centre International de Recherche en Infectiologie, Inserm, U1111, Université Claude Bernard Lyon 1, CNRS, UMR5308, Ecole Normale Supérieure de Lyon, Univ. Lyon, Lyon, France; ^5^Service de Chirurgie Orthopédique, Hôpital de la Croix-Rousse, Hospices Civils de Lyon, Lyon, France; ^6^Pherecydes Pharma, Romainville, France; ^7^Service de Chirurgie Plastique et Reconstructrice, Hôpital de la Croix-Rousse, Hospices Civils de Lyon, Lyon, France; ^8^Service de Maladies Infectieuses, Centre Hospitalier Metropole Savoie, Chambéry, France; ^9^Pharmacie, Hôpital de la Croix-Rousse, Hospices Civils de Lyon, Lyon, France; ^10^Institut des Agents Infectieux, Laboratoire de Bactériologie, Centre National de Référence des Staphylocoques, Hôpital de la Croix-Rousse, Hospices Civils de Lyon, Lyon, France

**Keywords:** prosthetic joint infection, bacteriophage, phage therapy, hydrogel, megaprosthesis

## Abstract

Infection is the most dramatic complication in patients with knee megaprosthesis. Its management is more complex in comparison with patients with primary arthroplasty, with a high risk of relapse. Lytic bacteriophages are considered to have a high potential in patients with prosthetic joint infection as it has been demonstrated that they have a synergistic anti-biofilm activity with antibiotics. The Defensive Antibacterial Coating (DAC®) hydrogel is a hydrogel available in the market that has been designed to prevent the adherence of bacteria on a prosthetic joint and to have the ability to transport and release anti-bacterial substances such as antibiotics. We report here the case of a patient with a catastrophic relapsing *Staphylococcus aureus* knee megaprosthesis infection without prosthesis loosening. We firstly perform phage susceptibility testing of the patient's strain to select an active cocktail, under the supervision of the French health authority. Then, we performed, as salvage therapy, a debridement and implant retention procedure with application of a selected cocktail of bacteriophages that was prepared extemporaneously within the DAC® hydrogel. A free flap for soft tissue coverage was required and empirical antibiotic treatment was started immediately after the surgery. Unfortunately, at 5 days after the surgery, while the local aspect of the surgical site was favorable, the patient developed myocardial infarction which required emergency stenting and dual antiplatelet therapy that were rapidly associated with bleeding at the surgical site, leading to a new prosthesis exposition. As a consequence, a transfemoral amputation was finally performed several months later. We also evaluated *in vitro* the impact of DAC® hydrogel on bacteriophage activity and showed that the selected phages were released very rapidly from the DAC® hydrogel, and then their titers were stable for at least 6 h. This case demonstrated the feasibility of the use of bacteriophages within a hydrogel to treat patients for knee megaprosthesis infection during a debridement procedure. The implementation requires identification of the pathogen before the debridement in order to perform phage susceptibility testing of the patient's strain and to identify a hospital pharmacist who will accept to do the preparation and to take the responsibility of the magistral preparation.

## Introduction

Knee megaprosthesis is used for patients with bone cancer or trauma that requires distal femur resection (1). Infection, which occurs in 3–40% in such patients, is one of the most terrible complications (2, 3). Its management is more complex in comparison with patients with primary arthroplasty as: (i) the “Debridement Antibiotics and Implant Retention” (DAIR) procedure is potentially associated with a higher rate of failure and (ii) one- or two-stage exchange is associated with higher morbidity and loss of function, especially if there is no loosening of the implants. DAIR is usually contraindicated in patients with chronic prosthetic joint infection (PJI) or in patients with PJI with prosthesis exposition.

Lytic bacteriophages rapidly kill *in vitro* specifically the targeted bacteria and self-replicate in an exponential and self-sustained reaction (4). They are considered to have a high potential in patients with PJI as it has been demonstrated that they have a synergistic anti-biofilm activity with antibiotics (5). In a few patients with relapsing chronic PJI for whom explantation was not possible, we previously performed DAIR and used a selected cocktail of bacteriophages that was injected into the joint as compassionate therapy, with a good clinical response (6). This approach is not a simple option for patients with infected knee megaprosthesis, especially in the case of prosthesis exposition that require soft tissue coverage. Indeed in this critical clinical situation, the surface of the infected joint is large, and there is no anatomical joint to contain the phages administered during DAIR surgery.

The Defensive Antibacterial Coating (DAC®) hydrogel (Novagenit, Mezzolombardo, Italy) is a hydrogel composed of two bioresorbable polymers (hyaluronic acid and poly-lactic acid) and that has been designed to prevent the adherence of bacteria (that are usually attracted by the hydrophobic surface of the implant) and to have the ability to transport and release anti-bacterial substances such as antibiotics. In a prospective observational multicenter study in patients for whom primary arthroplasty or prosthesis revision was performed, the use of the DAC® hydrogel was associated with a significant reduction of the rate of post-operative infection (7). In patients with PJI, two studies with a limited number of patients revealed that the use of the DAC® hydrogel during a one- or two-stage exchange may provide better infection control (8, 9).

We report here the case of a patient with a catastrophic relapsing *Staphylococcus aureus* knee megaprosthesis infection. The patient presented with prosthesis exposition, fistula, and purulent discharge, but without prosthesis loosening. We performed, as salvage therapy, a DAIR with the application of a selected cocktail of lytic bacteriophages within the DAC® hydrogel (as magistral preparation) after susceptibility testing of the phages against the patient's strain, and we finally performed a free flap for soft tissue coverage. We also evaluated the impact of DAC® hydrogel on bacteriophage activity.

## Case Description

**Figure 1:**
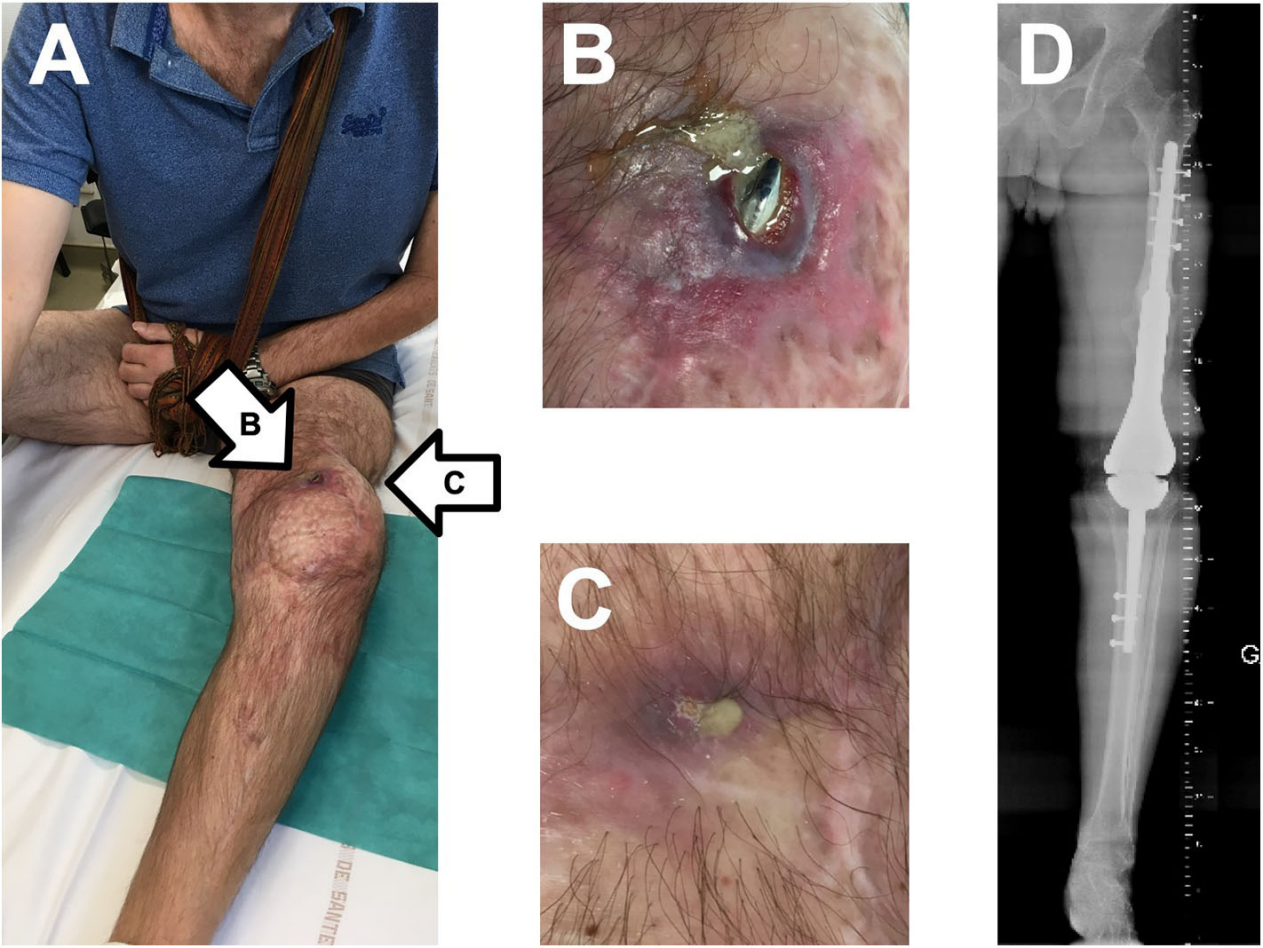
Clinical and X-ray status of the patient at baseline, with two fistulas regarding the femoral part of the megaprosthesis **(A)**, with purulent discharge and prosthesis exposition **(B,C)**, and without prosthesis loosening on X-ray **(D)**.

A 49-year-old man had a past history of trauma in 2012 with right scapula fracture, sternoclavicular luxation complicated by brachial plexus palsy, and open left distal femoral fracture. A knee megaprosthesis was used for reconstruction in 2013. As the patient developed skin and knee extensor necrosis, patellectomy and gastrocnemius skin and soft tissue flap were performed. In 2015, a multidrug-resistant *Staphylococcus epidermidis* PJI was diagnosed, and a two-stage exchange of the megaprosthesis was performed. Unfortunately, in 2016, a purulent discharge appeared. As methicillin-susceptible *S. aureus* (only resistant to penicillin) grew in culture from the discharge sample, but also from the puncture of an abscess in close contact with the prosthesis, clindamycin was prescribed as suppressive therapy. A new change of the megaprosthesis was considered to be not feasible, and the patient refused transfemoral amputation due to the terrible functional consequences as he still had the right brachial plexus palsy, making it impossible to walk with crutches. Finally, the patient developed two fistula (Figure 1A), with purulent discharge and prosthesis exposition (Figures 1B,C), without prosthesis loosening on X-ray (Figure 1D). We proposed, as salvage therapy, to perform a DAIR with local application of a selected cocktail of lytic bacteriophages under the supervision of the French National Agency for Medicines and Health Products Safety (ANSM) and in collaboration with the hospital pharmacist. Indeed phage therapy is not yet approved by the European Medicines Agency but “compassionate” use is however possible in France, under the supervision of ANSM, if the patient's status matches with article 37 of the Declaration of Helsinki, i.e., if proven interventions do not exist or if other known interventions have been ineffective (10). The final mix of bacteriophages has to be performed extemporaneously, under the responsibility of the hospital pharmacist, as this preparation becomes a “compounded” drug product, also called “magistral” preparation in Europe. In this particular case, the application of the mix of bacteriophages in a liquid formulation was complex as the infection was not limited to the joint but also concerned a large part of the femoral compartment of the megaprosthesis. Moreover, as the patient also had a large skin and soft tissue defect, with previous local flap, we planned to perform a free deep inferior epigastric perforator (DIEP) flap (i.e., taking skin and soft tissue from the abdomen to cover the megaprosthesis) (11). Considering all these elements, a carrier such as a gel was essential for phage application to keep the phages at the implant surface during the skin and soft tissue coverage. We proposed to use the DAC® hydrogel, which is available in the market and usable for patients with PJI.

## Materials and Methods

### Phages

The two phages, PP1493 and PP1815 [both *Caudovirales* (tailed bacteriophages)*, Herelleviridae* family], administered to the patient were selected from the Pherecydes phage bank. These bacteriophages, which were still in a development process, were not yet approved as drugs. Although the manufacturers followed the same processes as those established by the Good Manufacturing Practice (GMP) guidelines, they were produced in a research and development (R&D) laboratory (not GMP). The ANSM carefully reviewed the quality control tests applied to these batches, in collaboration with the hospital pharmacist and before the salvage therapy.

### Phagogram

The efficiency of these bacteriophages against the patient's strain was tested using the plaque assay to calculate the efficiency of plating (EOP) score and looking at the impact of the phages on the bacterial growth kinetics, hereafter referred to as kinetics assay. Plaque assay was based on the visualization of bacterial lysis when serial 10-fold dilutions of phages were spotted on solid medium containing either the patient's strain or the reference strain (spot plaque assay). When plaque-forming units (PFU) were observed, the EOP score was calculated by dividing the phage titer on the patient's strain by the phage titer on its reference strain showing the highest titer. The closer to 1 the score is, the more efficient the phage is. For the kinetic assay, the patient's strain was inoculated in a 96-well plate at a starting concentration of 1 × 10^6^ colony-forming units/ml with or without phages. The activity of each phage was tested individually at three different concentrations to obtain theoretical multiplicities of infection (MOI, ratio of phage/bacteria) equal to 1, 10, and 100 phages per bacteria and classified as low, intermediate, or high MOI. Bacterial growth was monitored over time by measuring the OD_600nm_.

### Impact of DAC® Hydrogel on Bacteriophage Activity

The suspension of phages was prepared by diluting 1 ml of each phage in 4 ml of water for injection (WFI). Then, 5 ml was added to the DAC® powder. Once homogenous, the hydrogel containing the phages was incubated for 10 min at room temperature. Once it turned solid, it was transferred into 10 ml of Dulbecco's phosphate-buffered saline (DPBS) and incubated at 37°C for 6 h. The phage titers were controlled in the 5-ml dilution (before powder addition) as well as in the DPBS at T_0_, T_0.5h_, T_1h_, T_2h_, T_4h_, and T_6h_.

## Results

### Phagogram

**Figure 2:**
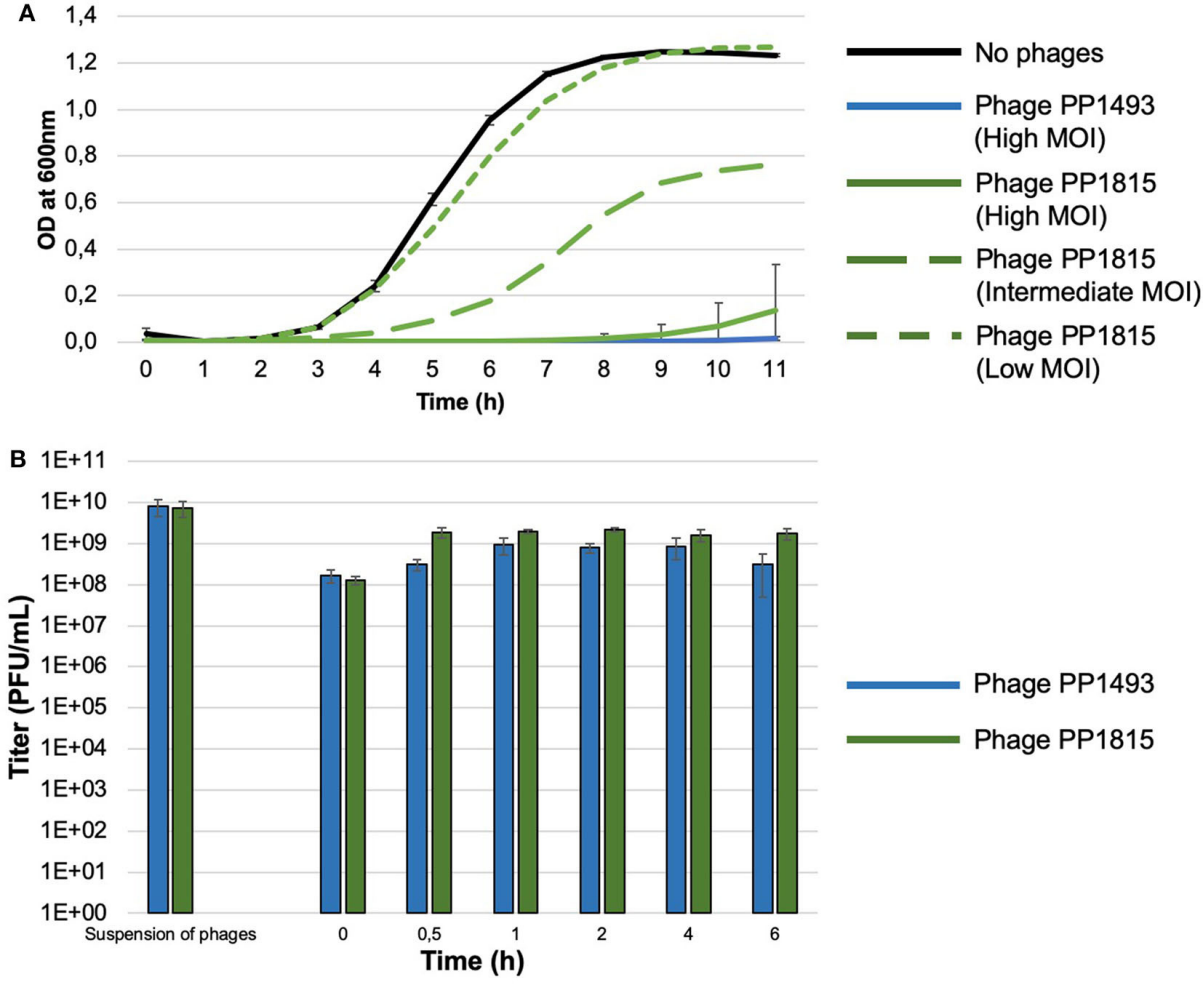
**(A)** Kinetic assay of phages PP1493 and PP1815 on a patient's strain at different multiplicities of infection (MOI). The X-axis represents the time and the Y-axis indicates the OD at 600 nm. The patient's strain growth without phage is represented with a full black line and with PP1493 with a blue line, no bacterial growth was observed whatever the MOI (only the high MOI is represented); with PP1815 (green lines), the bacterial growth was MOI dependent, with inhibition of the bacterial growth only at high MOI. **(B)** Impact of DAC® hydrogel on bacteriophage activity. The X-axis represents the time in hours and the Y-axis indicates the titer in PFU/ml. PP1493 is shown in blue, and PP1815 is shown in green.

The EOP assay revealed that phage PP1493 was active and very efficient on the patient's strain with visualization of PFU (EOP score of 6.4 × 10^−1^). The PP1815 phage was also active, with a partial bacterial lysis of the lawn where the phages were spotted. However, no PFU was observed. The minimum concentration of the spotted phages leading to the spot partial lysis was 5.09 × 10^5^ PFU/ml. In the kinetic assay, we observed a complete inhibition of the bacterial growth with PP1493 whatever the phage concentration was. For PP1815, the highest phage dose (1 × 10^9^ PFU/ml, corresponding to “high” MOI) also led to a total control of the bacterial growth, while the intermediate phage dose (1 × 10^8^ PFU/ml, corresponding to “intermediate” MOI) led to a partial control of the bacterial population, and the lowest phage dose (1 × 10^7^ PFU/ml, corresponding to “low” MOI) had no effect (Figure 2A). Even if PP1815 seemed to be less active than PP1493, we concluded that both of them were active against this *S. aureus* strain and have to be mixed to avoid the acquisition of phage resistance.

### Impact of DAC® Hydrogel on Bacteriophage Activity

PP1493 and PP1815 were diluted within WFI, which is recommended by the DAC® hydrogel supplier. Before the DAC® powder addition, PP1493 and PP1815 were at 8.0 × 10^9^ and 7.3 × 10^9^ PFU/ml, respectively. In the DPBS, upon transfer, the phage titers were 1.7 × 10^8^ and 1.3 × 10^8^ PFU/ml, respectively. Between T_0.5h_ and T_6h_, the titers ranged between 3.1 × 10^8^ and 9.3 × 10^8^ PFU/ml for PP1493 and between 1.6 × 10^9^ and 2.2 × 10^9^ PFU/ml for PP1815, respectively (Figure 2). These results indicated that PP1493 and PP1815 were released very rapidly from the DAC® hydrogel, and then their titers were stable for at least 6 h (Figure 2B).

## Diagnostic Assessment, Therapeutic Intervention, Follow-Up, and Outcomes

**Figure 3:**
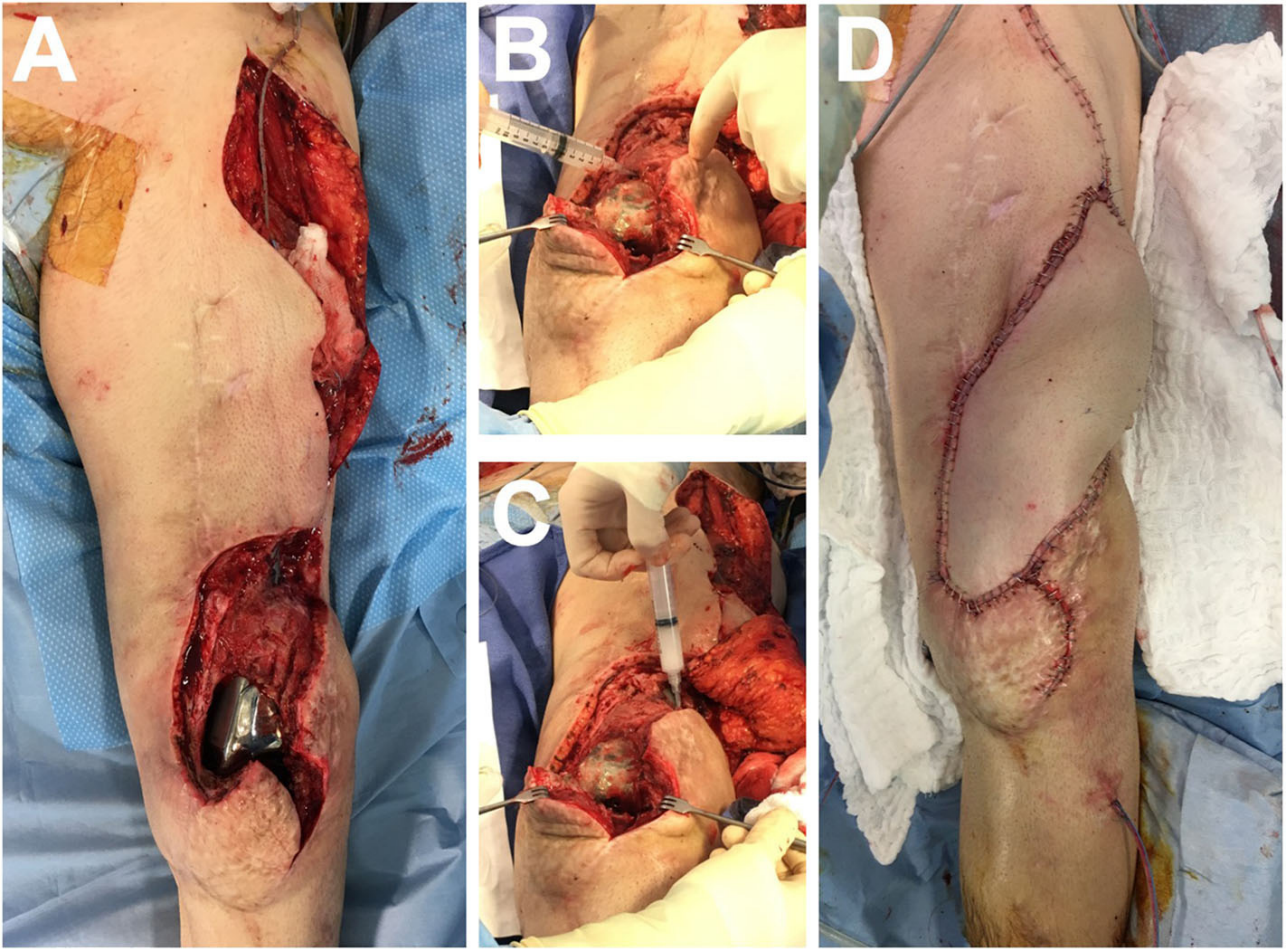
Peroperative pictures after the “Debridement Antibiotics and Implant Retention” **(A)**, during the application on the megaprosthesis surface of the magistral preparation containing the phages within the DAC® hydrogel **(B,C)**, and skin and soft tissue coverage with the deep inferior epigastric perforator free flap **(D)**.

We planned the therapeutic intervention under the supervision of the ANSM, and the patient signed a written consent. Two vials containing 1 ml of 10^10^ PFU/ml suspension of each bacteriophage in DPBS were received by our hospital pharmacist. Reconstitution of the DAC® hydrogel was performed according to the manufacturer's instructions. The prefilled syringe, containing 300 mg of sterile DAC® powder, was filled extemporaneously at the pharmacy, under sterile conditions, with a solution of 5 ml sterile water for injection, and 1 ml of each bacteriophage (10^10^ PFU/ml) was added instead of adding antibiotics. We performed open DAIR, which revealed, as expected, large suppuration in close contact to the femoral part of the prosthesis and into the joint. Several samples were taken for bacterial culture. Synovectomy and excision of infected tissue were performed, followed by a large irrigation with saline using a pulse lavage system. After the DAIR (Figure 3A), we applied the magistral phage preparation within the DAC® gel on the megaprosthesis surface (Figures 3B,C). Finally, the skin and soft tissue coverage with the DIEP free flap was performed (Figure 3D). Intravenous empirical antibiotic treatment with daptomycin (850 mg, one injection/day) and tigecycline (100 mg as initial dose, followed by 50 mg injected every 12 h) was started immediately after the surgery, pending the microbiological results, as the patient previously experienced a multidrug-resistant *S. epidermidis* infection. *S. aureus* grew in all microbiological peroperative samples, with the same antibiogram than that obtained before the surgery, except for a subpopulation of *S. aureus* that acquired erythromycin and clindamycin resistance. Unfortunately, at 5 days after the surgery, while the local aspect of the surgical site was favorable, the patient developed chest pain in relation with myocardial infarction. A coronarography was performed and revealed underlying atherosclerosis which, up to now, has been asymptomatic. An emergency stenting with dual antiplatelet therapy with salicylic acid and ticagrelor (a P2Y12 receptor antagonist) was required and prescribed. Bleeding at the surgical site rapidly occurred. At 25 days after the surgery, the free flap was perfectly integrated (without any sign of necrosis), but the bleeding persisted though the scar and led to a new prosthesis exposition. A local debridement, performed 1 month after the phage administration, revealed a hematoma under the free flap that communicated outside along the prosthesis exposition infection. A bacterial culture of the hematoma revealed superinfection with *Pseudomonas aeruginosa, Achromobacter* spp., and *Proteus mirabilis* in culture. No *S. aureus* grew in culture. Daptomycin was continued and tigecycline was replaced with ceftazidime, ciprofloxacin, and rifampin. As a discharge persisted, a new debridement was performed at 1 month later, without any bacteria in culture. Unfortunately, a new prosthesis exposition occurred and the patient decided to completely stop the antimicrobial therapy. At 1 year after the phage administration, a transfemoral amputation was finally performed, whereas the prosthesis exposition persisted, with a discharge. During amputation, the surgical samples revealed a polymicrobial infection with anaerobic flora, *Streptococcus anginosus, Finegoldia magna, P. mirabilis*, and *S. aureus* in culture (only resistant to penicillin and erythromycin; this latter strain was not genetically related to the first isolate as it belonged to the clonal complex 398, whereas the first strain belonged to the clonal complex 30). A pathology analysis of the bone did not reveal infiltration by inflammatory cells.

## Discussion

The risk of infection after the implantation of megaprosthesis is particularly high, especially due to the accumulation of several risk factors such as iterative past surgeries, extended incision, duration of surgery, implant's surface size, and chemotherapy or radiotherapy in oncologic patients. A conservative approach is a huge challenge in patients with infection of knee megaprosthesis without loosening. Indeed its management is considerably more complex in comparison with the management of primary prosthesis. The DAIR procedure followed with the administration of systemic antibiotics is a therapy generally offered to patients with acute or late acute PJI. Unfortunately, DAIR frequently fails in patients with knee megaprosthesis infection, at least in part due to the persistence of the pathogen on the implant's surface. The use of adjuvant agents that could have local anti-biofilm activity during the DAIR procedure seems to be of importance to control the infection in such patients and could facilitate the success of a potential subsequent suppressive antibiotic treatment (1–3, 12–20).

Bacteriophages are candidates to be used locally in patients with PJI to target the biofilm. Several past and recent data demonstrated that bacteriophages have an antibiofilm activity (21–23). The antibiofilm activities of the two phages used to treat the present case were also evaluated *in vitro* in a publication from our group. The activity of these two bacteriophages against the biofilm-embedded *S. aureus* was dose dependent. In addition, synergistic effects were observed when the bacteriophages were combined with antibiotics used at the lowest concentrations (5).

The administration of bacteriophages in patients with knee megaprosthesis is also conditioned by the use of an adequate dosage form that could cover the implant surface and deliver the phages locally. The gel formulation could perfectly fulfill these conditions, but it is important to demonstrate the stability of the phages within the gel and to evaluate its capacity to release phages. The DAC® gel is of interest as it is a CE-marked medical device approved to act as a physical barrier against bacterial colonization of the implant surface. Moreover, this hydrogel could be mixed with a bioactive agent, such as antibiotics, that could complement the gel's primary function. The manufacturer notifies that including a bioactive agent has to be taken at the surgeon's discretion, in the best interest of the patient under treatment. Finally, this gel has been used in several previous studies (7–9).

We demonstrated the *in vitro* activity of the phages on the patient's strain. Although PP1815 was only active at high MOI, according to the kinetic assay, it has been estimated that, thanks to the debridement, the adequate ratio of phage/bacteria could be achieved during the surgery. We also showed that phages can be released from the DAC® hydrogel and that it has no major impact on PP1493 and PP1815 activity. The association of DAC® hydrogel and bacteriophages seemed compatible; thus, we used purified selected phages into the DAC® hydrogel to treat this patient.

Unfortunately, post-operative myocardial infarction (that was not considered as a phage-related serious adverse event as the patient had previous asymptomatic atherosclerosis lesions) led to the formation of a hematoma under the free flap with important bleeding and prosthesis exposition, with secondary infection, and finally with the performance of an amputation. We do not think about a putative interaction between the antiplatelet drugs and the phages administered locally as the antiplatelet treatment was prescribed days after the phage administration and as bleeding is quite common with these drugs if they are prescribed after a surgery. During the amputation at 1 year after the phage administration, whereas the patient stopped all antibiotics for several months, *S. aureus* was again detected in culture but belonged to another clonal complex. It would be a new contamination of the exposed prosthesis, with a different *S. aureus* strain.

Concerning the safety of local phage administration within the gel, the patient developed myocardial infarction, with underlying atherosclerosis that was not known before surgery. We then observed the occurrence of hematoma that led to new prosthesis exposition, in relation with the prescribed antiplatelet treatment. These “adverse events” were not considered to be in relation with the phage administration.

This case demonstrated the practical feasibility of the use of bacteriophages within a hydrogel to treat patients for knee megaprosthesis infection during a DAIR procedure. The implementation requires identifying the pathogen before the DAIR, performing phage susceptibility testing of the patient's strain on the supervision of ANSM, and identifying a hospital pharmacist who will accept to do the preparation and to take the responsibility of the magistral preparation.

## Perspective

This is a potentially innovative approach to target the biofilm in patients with megaprosthesis knee infection. However, a prospective study including patients with such infection is complex to set up as there are some heterogeneity between the type of megaprosthesis, the clinical presentation of the infection, and the type of pathogen involved. An animal model of PJI demonstrating the microbiological and the clinical response to this therapeutic approach could be the next step.

## Data Availability Statement

The raw data supporting the conclusions of this article will be made available by the authors, without undue reservation.

## Ethics Statement

The studies involving human participants were reviewed and approved by Ethic committee of Hospices Civils de Lyon. The patients/participants provided their written informed consent to participate in this study.

## Author Contributions

TF managed the patient, directly interacted with the French Health authority and the companies to propose the study plan and to obtain the bacteriophages and the gel, and wrote the manuscript. CP and CF performed the preclinical stability study. GL prepared the phages. CB, JC, and SL performed the surgery. FL and CK performed the microbiological diagnosis. EF also participated in patient care. All authors participated in the literature review and the improvement of the manuscript.

## Conflict of Interest

CP and CF are employed by the commercial company Pherecydes Pharma. The remaining authors declare that the research was conducted in the absence of any commercial or financial relationships that could be construed as a potential conflict of interest.
